# Solitary Cerebellar Toxoplasmosis as the First Presentation of HIV Infection: A Case Report and Review of Literature

**DOI:** 10.7759/cureus.70456

**Published:** 2024-09-29

**Authors:** Alaa N Turkistani, Tala AlSindi, Mohammed Homoud, Fahad Alghamdi, Saleh S Baeesa

**Affiliations:** 1 Neurosurgery, King Faisal Specialist Hospital and Research Centre, Jeddah, SAU; 2 Neurosciences, King Faisal Specialist Hospital and Research Centre, Jeddah, SAU; 3 Pathology, King Faisal Specialist Hospital and Research Centre, Jeddah, SAU

**Keywords:** aids (acquired immunodeficiency syndrome), cerebellar toxoplasmosis, cns parasitic infection, human immunedeficiecy virus (hiv) infection, toxoplasmosis gondii

## Abstract

Toxoplasmosis is the most common space-occupying lesion in HIV-infected patients that typically presents as a space-occupying lesion in the supratentorial region, often manifesting as focal neurological deficits. Infratentorial toxoplasmosis is extremely rare, with a few reported cases in the literature.

Here, we are reporting a 53-year-old healthy female presented with isolated single cerebellar toxoplasmosis as a first manifestation of HIV infection. The patient underwent suboccipital craniotomy and tumor excision, and the subsequent histopathological exam revealed toxoplasma protozoan.

While cerebral toxoplasmosis is common in HIV-infected patients, cerebellar involvement is rare and requires prompt diagnosis for effective treatment. A high index of suspicion is emphasized to prompt early diagnosis and initiation of therapy.

## Introduction

Toxoplasmosis is considered the most encountered space-occupying lesion in HIV-infected patients [1]. Most individuals with primary infections are subclinical, and the others only show mild illness in the immunocompetent. However, in immunocompromised individuals, such as acquired immunodeficiency syndrome (AIDS), the disease becomes severely symptomatic, especially when the cluster of differentiation 4 (CD4) count declines to less than 200 cells/mm^3^, moreover with a CD4 count of fewer than 50 cells/mm^3^ [2]. Toxoplasma encephalitis is an established complication in HIV patients, presented with a space-occupying lesion in the supratentorial area, usually manifesting as a focal neurological deficit [3,4]. Infratentorial toxoplasmosis is extremely rare, with a few reported cases in the literature review [5-9]. We report a case of isolated cerebellar toxoplasmosis as the first manifestation of HIV in a previously healthy patient who responded successfully to treatment.

## Case presentation

A 53-year-old woman with type 2 diabetes mellitus (DM2) presented with a one-month history of episodic headaches, frequent vomiting, blurred vision, and ataxic gait, leading to multiple falls. She also reported night sweats, loss of appetite, and lost about 8 kg over six weeks. There were no additional neurological symptoms, history of alcoholism, use of illicit drugs, or smoking. She has been married for five years and denied any sexual activity outside of marriage. She had no family history of a similar illness.

On examination, she had normal vital signs. She was alert, conscious, and oriented with normal cognitive function. Neurological examination revealed left-sided dysdiadochokinesia, positive finger-to-nose and heel-to-shin tests, and an ataxic gait towards the left side with a positive Romberg test. There was no cranial neuropathy, and motor and sensory functions were normal in all extremities and normal reflexes. 

**Figure 1:**
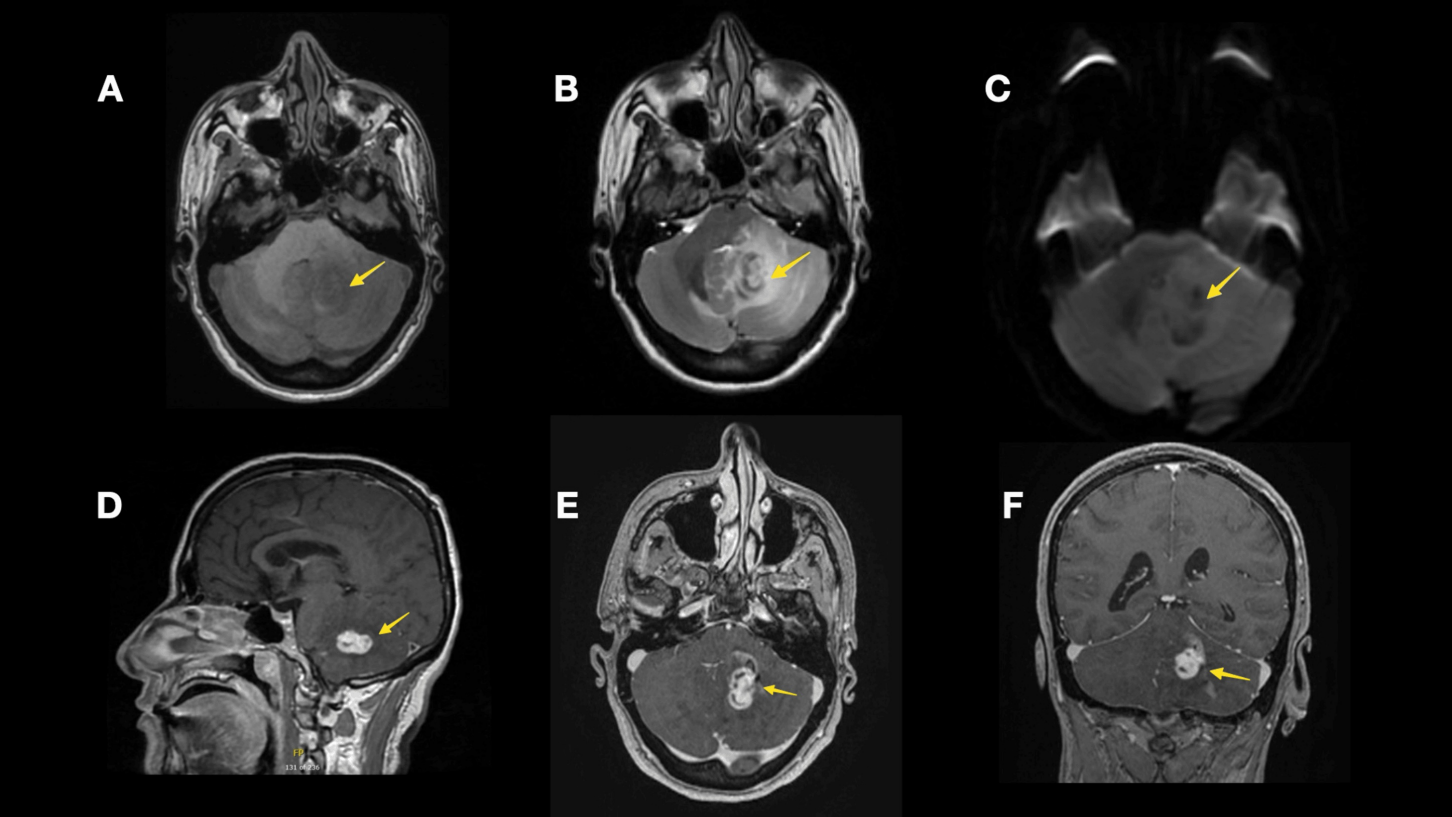
Brain MRI scan images Brain MRI scan revealed an isodense deep left cerebellar hemisphere measuring 2.3 x 1.4 x 1.4 cm lesion (yellow arrow) in the axial T1-WI (A), with marked peri-lesional edema in the axial T2-WI (B). The lesion has no fluid restriction on DWI (C). The lesion demonstrated a homogenous enhancement following intravenous contrast administration in the sagittal (D), axial (E), and coronal (F) views.

Magnetic resonance imaging (MRI) scan showed left cerebellar mass (2.3 × 1.4 × 1.4 cm) with a low signal on T1-weighted images and a high signal on T2-weighted images. No restricted diffusion was noted in the diffusion-weighted images. The mass shows avid enhancement on post-contrast images, with surrounding edema exerting a mass effect on the fourth ventricle (Figure 1). 

Routing laboratory tests were within normal range, including complete blood count, electrolytes, renal and liver profiles, and coagulation parameters. A provisional diagnosis of metastasis, primary tumors, or infectious process was made, and the patient consented to surgery. Suboccipital craniotomy was performed, and exposure was guided by neuronavigation. The microscope was introduced to the field, and a tumor was encountered, which looked intraoperatively like pale greyish soft tissue. Multiple samples were taken for frozen sections; however, none was conclusive. Resection using an ultrasonic aspirator was started, and all dominations of the surgical cavity were inspected to detect residual tumors.

**Figure 2:**
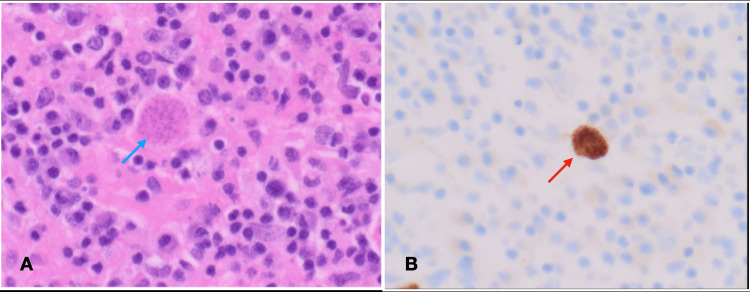
Histopathology images Histopathological photograph (A): hematoxylin stain showing intracytoplasmic microorganisms (bradyzoite) (blue arrow). magnification 400x. (B): Immunohistochemistry positive for toxoplasma (red arrow).

Histopathological exam showed necrotizing lesions with a mixed inflammatory infiltrate, including lymphocytes, plasma cells, and macrophages, along with tachyzoites and bradyzoites of *Toxoplasma gondii* within cysts and accessible in the necrotic areas as shown. These lesions are surrounded by reactive gliosis and are morphologically associated with toxoplasma protozoan (Figure 2).

After the cerebellar toxoplasmosis diagnosis, the infection disease services investigated the patient. They revealed that HIV 1-2 antibody screen and HIV 1-2 confirmatory, which returned reactive, with ABS Helper T CD4 cell count of 448.0 (normal range; 564.0-1721.0 10^6^/L), Helper T CD4 of 13% (normal range; 31-53%), and a CD4/CD8 0.19 (1.04-2.62 10^6^/L), while her viral load was 2134207. Toxoplasmosis immunoglobulin (Ig)G Antibody was 111.10 IU/mL (< 1.0 IU/mL nonreactive, 1.0 to 2.99 intermediate, 3.0 and above indicative of toxoplasmosis infection), and Hepatitis A, B, and C were negative. 

The patient had an uneventful recovery and started on antitoxoplasmosis medications, including pyrimethamine, sulfadiazine, and leucovorin, as well as antiretroviral therapy (Biktravy®, one tablet orally once a day) for HIV and was discharged home.

**Figure 3:**
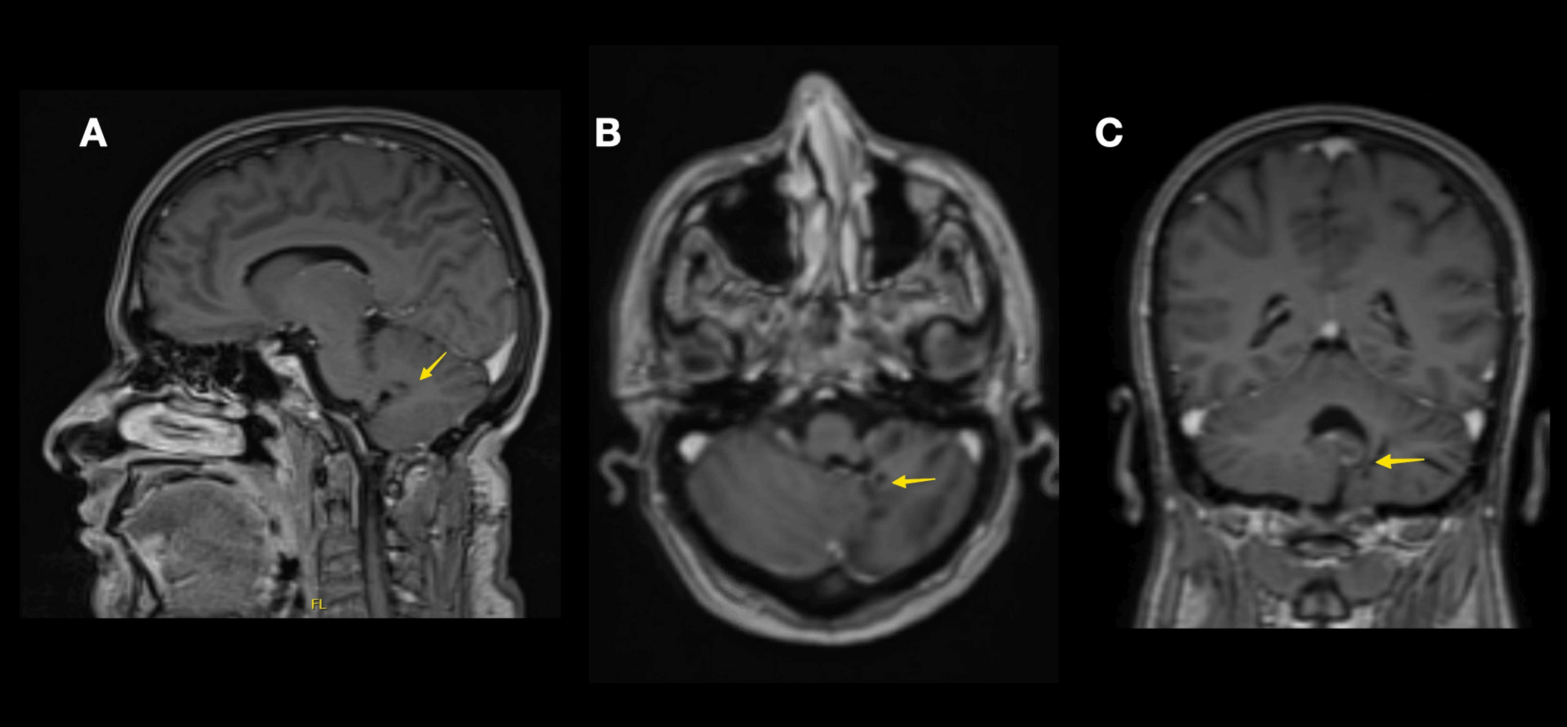
Brain MRI (with contrast) scan obtained after one year Brain MRI with contrast scan at one-year follow-up in sagittal (A), axial (B), and Coronal (C) views revealed interval resolution of previously noticed lesion of nodular enhancement (yellow arrow) within the left cerebellar hemisphere.

Upon follow-up, the patient started on antiretroviral medications, and her last CD4 cell count was 13. Neurological assessment revealed marked clinical improvement in her symptoms, mild gait ataxia, and no other abnormalities. She completed a two-month course of antibiotics. Repeated brain MRI revealed interval resolution of previously noticed focus of nodular enhancement within the left cerebellar hemisphere (Figure 3).

## Discussion

Toxoplasmic encephalitis (TE) is a well-recognized major central nervous system (CNS) complication in patients with AIDS [10,11]. TE presents as single or, more commonly, multiple brain abscesses, with a propensity to the deep gray matter structures or the blurred gray/white matter junction. The basal ganglia represent the most common location (48%), the frontal lobe (37%), and the parietal lobe (37%). To a lesser extent, it might occur in the occipital lobe (19%), temporal lobe (18%), and brain stem/cerebellum (5-15%), being the least for which it requires a high index of suspicion [12,13].

Clinical presentation varies according to the location of involvement. Headache and focal neurological deficit have been reported as the most common manifestations in TE, with or without fever. Accompanying cerebral edema will manifest as confusion and lethargy, which can evolve into a coma [14,15]. Initial neuroimaging is essential to start empirical therapy. Magnetic resonance imaging (MRI) is the preferred modality for evaluating brain lesions since its sensitivity is higher than that of a CT scan in the case of a diagnosis of cerebral toxoplasmosis [16,17]. In CT scan, it either appears as typical ring-enhancing lesions with perilesional edema (44%-82%), nodular-enhancing lesions with perilesional edema (3-33%), or atypical non-enhancing lesions with expansive effect (6%-20%) can be observed [12,18]. There are two specific but non-sensitive signs described to be associated with TE. A postcontrast CT or T1-weighted MRI showing a ring-shaped zone of peripheral enhancement, with a small eccentric nodule along the wall, is called an ‘eccentric target sign’ although unusual (<30% of cases) and considered a specific sign for TE. Secondly, we have the (concentric target sign) on MRI T2-weighted images, described as concentric alternating zones of hypo- and hyperintensities [19]. Even though certain radiological features support primary CNS lymphoma (PCNSL) over TE (i.e., single lesion, periventricular lesion, subependymal spread, and homogenous rather than ring enhancement), there is still an overlap between the two conditions. Hence, the clinical and radiological features cannot differentiate PCNSL from TE [18].

For that, tissue-based diagnosis visualizing the tachyzoites, obtained by stereotactic or open brain biopsy, is the standard procedure to diagnose focal brain lesions, considered diagnostic and therapeutic. The most common differential diagnoses of a focal brain lesion in AIDS patients are PCNSL (15%-28%), PML (21%-22%), and cerebral toxoplasmosis (19%-20%) [20]. CSF *T. gondii* polymerase chain reaction (PCR) assay has a moderate sensitivity but a high specificity and positive predictive value, especially if done within the first week. Diagnosis of cerebral toxoplasmosis can be established if CSF *T. gondii* PCR assay is positive, but a negative result does not exclude it. Nonetheless, performing PCR for all HIV patients who are suspected of having a cerebral infection is worthwhile, as this will help to avoid needless brain biopsy/surgery and allow early management [21]. When lumber puncture is contraindicated, *T. gondii* PCR assay in blood samples can be performed [22]. As cerebral toxoplasmosis usually causes no meningeal involvement, CSF analysis has no relevant value [23]. Another test to guide the differential diagnosis of space-occupying lesions in AIDS patients is the CD4 cell count. PCNSL, cerebral toxoplasmosis, and cerebral TB patients tend to have a CD4 cell count of 50 cells/mm^3^, below 100, and 200 cells/mm^3^, respectively [16,17].

Management of CNS toxoplasmosis with anti-toxoplasma therapy, including pyrimethamine plus sulfadiazine (P-S), demonstrates an 80% to 90% clinical and radiological improvement [24]. It is worth mentioning that the radiologic response to antitoxoplasma therapy lags behind the clinical response [12]. Outcomes vary from case to case, according to the severity of the initial neurological state and time of diagnosis and treatment administration. Starting treatment as early as possible is essential to prevent long-term neurological sequelae [17,25]. Treatment with pyrimethamine plus clindamycin (P-C) or trimethoprim-sulfamethoxazole (TMP-SMX) vs. treatment with (P-S) showed similar clinical and radiological response and drug compliance rates [26]. Steroids should only be used in case of significant mass effect or massive diffuse brain edema is present [16]. A review of the literature reveals only five cases of isolated cerebellar toxoplasmosis in HIV/AIDS patients, with our case being the sixth to be reported (Table 1).

**Table 1:** Literature review on isolated cerebellar toxoplasmosis in HIV/AIDS patients, including our case IgG: Immunoglobulin G.

Case No./ First author (year) (reference)	Age/Gender	Presenting symptoms	HIV status before diagnosis	Investigation Results	Management	Outcome
Gaggero et al. (2022) [5]	43Y/F	Headache and ataxia	Positive	Radiology: MRI brain: cerebellar enhancing mass with leptomeningeal spread and mass effect compression of the 4^th^ ventricle and mesencephalic duct	Intravenous broad-spectrum antibiotic therapy	The patient died quickly before any further diagnostic/therapeutic steps could be taken. Autopsy: Histological examination revealed multiple pseudocysts enclosing numerous hyperchromic corpuscles consistent with bradyzoites of Toxoplasma at the transition between the cerebellar granular layer and the molecular layer.
Pott et al. (2013) [6]	50Y/M	Stumbling, poor coordination, and falls	Unknown	Blood test: Toxoplasmosis IgG reactive and IgM non-reactive. (HIV-1) positive. CD4= 43 cells/mm^3^. Radiology: MRI brain: heterogeneous lesion with peripheral enhancement in the right cerebellar hemisphere, measuring 2.3x1.8 cm	Dexamethasone, sulfadiazine + pyrimethamine + folinic acid, ﬂuconazole, and trimethoprim + sulfamethoxazole.	Improvement after 14 days of hospitalization
Ibebuike et al. (2012) [7]	11 months/F	Developmental milestones deterioration and increased HC	Unknown	Blood test: HIV 1&2= Positive for both mother and child. CD4= 1,639X10^6^/l Radiology: CT brain: a ring-enhancing posterior fossa mass (7x4.5 cm) on the right side with compression of the 4^th^ ventricle causing hydrocephalus Histopathology: Toxoplasmosis.	Ventriculoperitoneal shunt insertion, then fresh specimen biopsy Antitoxoplasmosis therapy was started using sulfamethoxazole and trimethoprim.	Follow-up CT was performed 10 weeks later and showed a reduction in the size of the cerebellar mass and decompression of the 4th ventricle. The patient was well and alert.
Emeka et al. (2010) [8]	34Y/M	Right-sided weakness, gait disturbance and incoordination of the limbs.	Positive	Radiology: CT brain: a ring-enhancing lesion in the right lobe of the cerebellum without associated peri-lesional edema.	He received clindamycin, pyrimethamine, and pyridoxine.	He improved remarkably with a resolution of cerebellar signs.
Issakhanian et al. (2001) [9]	48Y/M	Vertigo, nausea, vomiting, and occipital headache.	Unknown	Radiology: MRI brain: hypointense lesion with some mass effect in the left cerebellum. The lesion showed ring enhancement after gadolinium injection. Blood test: HIV-1 negative. However, the CD4= 19/mm^3^. HIV-2 = positive Histopathology: A fragment of the cerebellar lesion showed gliosis focal necrosis. With cysts and numerous encysted tachyzoites, which were immunoreactive to antibodies against Toxoplasma gondii	Craniotomy and biopsy. Antitoxoplasmosis therapy was started with potent antiretroviral therapy.	Repeated imaging one year later showed a more minor cystic lesion.
Current case	53Y/F	Headache, blurring vision, vomiting, ataxia	Unknown	Radiology: MRI of the brain showed a left- enhancing cerebellar lesion. Blood test: HIV 1-2 antibody screen positive, CD4 =168 cells/mm, viral load was 2134207. Toxoplasmosis IgG Antibody =185.6 IU/ml, Histopathology: Necrotizing lesions with a mixed inflammatory infiltrate, including lymphocytes, plasma cells, and macrophages, along with tachyzoites and bradyzoites of *Toxoplasma gondii* within cysts	Suboccipital craniotomy and lesion resection	Repeated MRI of the brain revealed interval resolution of previously noticed focus of nodular enhancement

To the best of our knowledge, this is the ﬁrst reported case of isolated cerebellar toxoplasmosis of an HIV-infected patient in Saudi Arabia. Previously, there was one case reported in Latin America; two cases had been reported in Nigerian HIV-infected patients and two cases in South African patients. 

The ﬁrst case of cerebellar toxoplasmosis was reported in 2001 by Issakhanian et al. [9]. They described a case of a Nigerian man who had acute onset of occipital headache, vertigo, nausea, and vomiting; upon examination, he had gait ataxia and bilateral dysmetria. Noncontrast T1-weighted MRI revealed a hypointense lesion in the left cerebellum. After gadolinium injection, the lesion showed a ring enhancement sign. He underwent craniotomy for biopsy, and histopathology of the cerebellar lesion revealed toxoplasmosis. HIV-1 infection was suspected, but serological tests were negative multiple times. However, HIV-2 speciﬁc serology turned out to be positive, with a CD4 count of 19 mm. So, he was treated with anti-toxoplasmosis therapy. His repeat imaging studies, one year later, showed a smaller cystic lesion.

In 2010, Emeka et al. reported a case of a 34-year-old Nigerian man previously diagnosed with HIV/AIDS [8]. He presented with right-sided weakness, throbbing headaches, vertigo and vomiting, and right-sided cerebellar signs [8]. CT brain showed a ring-enhancing lesion in the right cerebellar hemisphere. He was managed as a case of cerebellar toxoplasmosis, and anti-toxoplasmosis therapy was started. Patient symptoms recurred with signs of cerebellar involvement due to poor compliance.

In 2012, Ibebuike et al. [7] reported a case of an 11-month-old South African infant presented with deterioration in developmental milestones and increased size of the head, and HIV 1 and 2 rapid screening tests were performed, which were reactive in both mother and child, with a CD4 count of 1,639 x 10^6^/L. Upon neurological assessment, the child was drowsy, with full and tense anterior fontanelle, horizontal nystagmus, cranial nerves II, III, and IV palsies, and right-sided hypotonia. CT brain revealed a ring-enhancing mass lesion in the right cerebellar hemisphere with secondary obstructive hydrocephalus, for which an urgent ventriculoperitoneal shunt was inserted. Then, she underwent elective posterior fossa decompression. The initial pathology report described a neoplastic process. Due to severe tissue necrosis, immunohistochemical stains were interpreted. Then, a fresh specimen was sent again and revealed the diagnosis of toxoplasmosis, and anti-toxoplasmosis therapy was started. Follow-up CT scan of the brain ten weeks after discharge showed a reduction in the size of the cerebellar mass and decompression of the 4th ventricle, along with clinical improvement.

In 2014, Pott et al. reported a case of a 50-year-old Latin American man presented with stumbling, poor coordination, falls, headache, and right-sided cerebellar signs [6]. Initial lab tests showed toxoplasmosis IgG reactive serology for human immunodeﬁciency virus 1 (HIV-1) was positive with a CD4 cell count of 43 cells/mm^3^. MRI revealed a heterogeneous lesion with peripheral enhancement to the contrast in the right cerebellar hemisphere, described as an eccentric target sign. A cerebellar toxoplasmosis diagnosis was made, and the patient started on anti-toxoplasmosis therapy. After 14 days of hospitalization, the patient’s general condition improved, with total remission of cerebellar symptoms. Repeated MRI of the brain shows a reduction of the lesion.; thus, he was discharged with outpatient follow-up.

In 2022, Gaggero et al. reported a case of a 43-year-old South American female previously known to have HIV on antiretroviral therapy, presented with headache and ataxia [5]. MRI revealed a cerebellar enhancement with leptomeningeal spread and mass effect compression of the 4th ventricle and mesencephalic duct, which is compatible with an infectious hypothesis. So, intravenous broad-spectrum antibiotic therapy was started, and it initially showed benefits. Yet, the patient’s neurological condition worsened again, and she died quickly before any further steps could be taken. An autopsy revealed a dark red area on the cerebellar surface corresponding, at sagittal and horizontal cutting, to a 5 cm large reddish zone involving both cerebellar hemispheres and the vermis. Histological examinations showed multiple abscesses with widespread parenchymal necrosis, configuring a necrotizing cerebellitis. At higher magnification, multiple pseudocysts enclosing numerous hyperchromic corpuscles consistent with bradyzoites of Toxoplasma were observed at the transition between the cerebellar granular layer and the molecular layer.

## Conclusions

Toxoplasmosis infection in HIV-infected patients is not an uncommon presentation, but the involvement of the cerebellum is rarely encountered, which makes such a condition clinically underestimated. We emphasize that, as presented in our case report, patients may present with cerebellar toxoplasmosis without being diagnosed with HIV/AIDS disease and may be the first manifestation of HIV/AIDS disease. Generally, toxoplasmosis infection carries a good prognosis when initiating early and appropriate antibiotics therapy, and a high index of suspicion is needed to prompt early diagnosis and initiation of treatment.
